# Biologic drugs in the treatment of chronic inflammatory pulmonary diseases: recent developments and future perspectives

**DOI:** 10.3389/fimmu.2023.1207641

**Published:** 2023-06-02

**Authors:** Jacek Plichta, Piotr Kuna, Michał Panek

**Affiliations:** Department of Internal Medicine, Asthma and Allergy, Medical University of Lodz, Lodz, Poland

**Keywords:** biologic drugs, monoclonal antibodies, asthma, COPD, IPF, pulmonary sarcoidosis

## Abstract

Chronic inflammatory diseases of the lung are some of the leading causes of mortality and significant morbidity worldwide. Despite the tremendous burden these conditions put on global healthcare, treatment options for most of these diseases remain scarce. Inhaled corticosteroids and beta-adrenergic agonists, while effective for symptom control and widely available, are linked to severe and progressive side effects, affecting long-term patient compliance. Biologic drugs, in particular peptide inhibitors and monoclonal antibodies show promise as therapeutics for chronic pulmonary diseases. Peptide inhibitor-based treatments have already been proposed for a range of diseases, including infectious disease, cancers and even Alzheimer disease, while monoclonal antibodies have already been implemented as therapeutics for a range of conditions. Several biologic agents are currently being developed for the treatment of asthma, chronic obstructive pulmonary disease, idiopathic pulmonary fibrosis and pulmonary sarcoidosis. This article is a review of the biologics already employed in the treatment of chronic inflammatory pulmonary diseases and recent progress in the development of the most promising of those treatments, with particular focus on randomised clinical trial outcomes.

## 1 Introduction

The classical definition of inflammation is “a response which arises in vascularised tissue upon exposure to infections and damaging stimuli, which recruits host defence cells to the site of exposure to eliminate the harmful agents”. The role of correctly functioning inflammation is that of an immediate response to the presence of pathogens at the site of a lesion. Inflammation constitutes a part of the innate immune response, the first line of the immune defence system (1). Normally, inflammation is an acute event which ultimately supports wound healing. Leukocytes migrate to the site of inflammation, remove the pathogens and support the process of tissue repair. However, in the event of dysregulation, inflammation can become chronic. Chronic inflammation is a detrimental process involving persistent, abnormal inflammation which ultimately leads to tissue damage and transformation (1). Chronic inflammation is the pathophysiological basis of a plethora of disease units, with several of the most prevalent and severe conditions affecting the respiratory system. Pulmonary chronic inflammation is one of the leading causes of mortality and significant morbidity worldwide. In 2019, 262 million active cases of asthma were estimated, causing 455 thousand deaths worldwide (2). Chronic obstructive pulmonary disease (COPD) is responsible for the third highest number of deaths worldwide, surpassed only by ischemic heart disease and malignant neoplasms. In 2015, COPD was estimated to affect around 174 million people worldwide, leading to approximately 3.2 million fatalities (2).

Common therapeutics for chronic inflammatory lung disease symptoms such as inhaled corticosteroids and beta-adrenergic agonists are effective and widely utilised. However, sustained long-term usage of these drugs is linked to increasingly severe side effects. To ensure continuing patient compliance required to properly manage these conditions, new pharmacological targets related to the pathophysiology of inflammation have to be considered and understood. An assortment of cell signalling pathways, related to a network of receptors and ligands have been linked to inflammation. Targeted inhibition of select elements within these pathways may serve as a superior approach to anti-inflammatory drug design.

Inhibiting specific pathways to manage chronic inflammation has many advantages over classical approaches to treatment, as the drugs target underlying pathophysiology of a given disease directly, instead of managing symptoms. Two main categories of therapeutic inhibitors are currently used to treat patients - small molecule drugs and biologic drugs. Two relatively recent subclasses of biologic drugs - peptide inhibitors and monoclonal antibodies show promise as therapeutics. Peptide inhibitor-based treatments have already been proposed for a range of diseases, including infectious disease (3, 4), cancers (5) and even Alzheimer disease (6), while monoclonal antibodies have already been implemented as therapeutics for a range of conditions. This review describes a selection of biologic inhibitors which are currently undergoing research and clinical trials as potential treatments for chronic inflammatory lung diseases.

## 2 Small molecule and biologic agents

Biological medications are a category which includes products such as vaccines, blood components, recombinant proteins or even somatic cells. The composition of biologics may include any combination of proteins, nucleic acids and sugars. Biologics can be derived from a variety of sources, such as animals, microorganisms or humans. The production of biologics frequently utilises cutting-edge biotechnological methods, and novel types of biological therapeutics such as cellular or genetic biologics constitute the frontiers of biomedical research. Unlike traditional drugs produced by chemical synthesis, with strictly defined structures, many biologics are heterogeneous mixtures of substances which may not be precisely defined. Biologics are highly specific and exhibit very low levels of toxicity, compared to small molecule drugs. However, biologics may display immunogenicity in some patients, unlike most small molecule drugs (7). The primary factor hindering the use of biologics is their enormous cost. Unlike small molecules, the structure of proteins is rife with complexities such as folding patterns or surface glycosylation. Thus, the manufacturing process of most biologics is highly complex. With currently available technologies, scaling up production and maintaining consistency between batches of biologics is challenging. Problems with heterogeneous post-translational modifications or discrepancies in protein conformation are common issues. Biologics tend to be sensitive to heat and microbial contamination, unlike chemically synthesised drugs. Finally, some patients may develop immune responses to biologics, causing a gradual loss of effectiveness and increase in immunogenicity, at unpredictable rates (8).

Small molecule drugs (SMs) are defined as low molecular weight (0.1 to 1 kDA) compounds capable of modulating biochemical processes *in vivo*, which can be used in disease diagnosis and management. Examples of SMs include diphenhydramine and aspirin. This category of drugs remains a useful and attractive option despite advances in the field of biologics for several reasons. The relatively simple chemical structure and low molecular weight of most SMs lead to pharmacokinetics and pharmacodynamics more calculable than those of biological drugs, and ultimately - less complicated administration protocols. The development, characterising and manufacturing of SMs also tend to be simpler than those of biologics. Due to the lower cost of raw materials and relative simplicity of the synthesis process, production costs of SMs may be a fraction of the cost of producing biologics. Lastly, SMs are usually highly stable molecules and can be administered orally, enhancing patient compliance, unlike antibodies and proteins, the current formulations of which must be administered intravenously. Due to all these factors, SMs are in most cases a more affordable option than biologics. What is more, SMs can pass through the cellular membrane and affect intracellular receptors, intranuclear targets and the nervous system. Outside of lysosomes and endosomes, delivering proteins to intracellular targets is difficult. Gene therapy may offer a solution to this limitation of biologics, but currently SMs remain the best choice for targeting most intracellular targets. However, as most SMs function by imitating effector molecules or through allosteric regulation of enzymes, not all biological processes are viable targets for SM-based regulation. What is more, several microbes and cancers exhibit resistance to SMs due to modifications in enzyme structure and chemistry, presence of efflux pumps or mutations rendering drug targets unrecognisable (9). A comparison of the main properties of SMs and biologics can be found in **Table 1**.

**Table 1:** A comparison of the main properties of small molecule drugs and biologic drugs.

Property	Small molecules	Biologics
Size	<1 kDa	1-200 kDa
Stability	Stable	Unstable
Structure	Simple	Complex
Specificity	Low	High
Primary administration route	Oral	Intravenous
Permeability	High	Low
Major metabolising enzymes	Oxidases, hydrolases, conjugating enzymes	Nucleases, peptidases, proteinases, hydrolases
Immunogenicity	No	Yes

Evidently, SMs and biologics are vastly different categories of drugs which vary substantially in many of their attributes. This is caused by their disparate physicochemical properties which affect the pharmacodynamics, pharmacokinetics, safety and efficacy and even manufacturing protocols of the drugs. SMs are simple chemical compounds, compared to biologics. Attributes such as stability, membrane permeability and pharmacological activity of SMs depend on their chemical composition. On the other hand, structural interactions within the tertiary structures of large therapeutic proteins are integral for their biological activity (7).

Due to their properties, SMs can be administered by most available routes, including the oral route. Indeed, oral bioavailability is one of the key advantages of SMs over biologics. Most biologics are polar and unable to permeate membranes. What is more, proteins tend to be vulnerable to enzymatic degradation rendering them ineligible for oral administration. Therefore, most biologics require systemic administration routes such as injections (7).

Pharmacodynamics are another key area of difference between SMs and biologics. Biologics tend to be much more highly specific than SMs. SMs bind to targets such as ligand-gated ion channels, receptor tyrosine kinases or G protein-coupled receptors (GPCRs) on both extra- and intracellular domains throughout the body. What is more, SMs are prone to disrupting biological processes through physical interactions with cellular components. As most SMs lack the strict specificity of biologics, they are liable to induce off-target effects. SMs frequently interact with cells or cellular components in undesirable ways, potentially leading to adverse effects. In contrast to SMs, biologics bind to their targets with high specificity, with a very low risk of off-target effects. However, the use of biologics is not without risk, as this category of drugs can impede physiological functions or even lead to life-threatening events by eliciting immunogenicity (8).

Aside from a small proportion of local-acting drugs, most drugs require being absorbed into systemic circulation to induce their effects. Most drugs are distributed to their sites of action through the blood circulatory system to be absorbed by cells, metabolised and eliminated. However, for the distribution of drugs with molecular masses over 10 kDa (which includes many biologics) the lymphatic system is equally important. These large drugs achieve transcellular transport primarily via processes such as phagocytosis or receptor-mediated endocytosis. Consequently, these large biologics exhibit adsorption, distribution, metabolism and excretion (ADME) characteristics different to those of SMs.

The differences include longer half-lives, smaller distribution volumes and longer times to reach peak concentration. As biologics interact with receptors with high affinities, elimination rates are directly correlated with binding efficiency. Therapeutic proteins are intracellularly metabolised by proteases and peptidases into amino acids, which are mostly reused by the body, with a small proportion excreted into feces or urine and eliminated. On the other hand, SMs are eliminated by non-targeted organs such as the kidneys, excreted in bile or feces or metabolised through cytochrome P450 metabolism (8). There exists an interesting misconception, that due to being produced by living cells, biologics require less stringent testing than SMs. In actuality, the manufacturing protocols of biologics often include quality control measures stricter than those required for SMs to avoid manufacturing errors. Such errors can potentially introduce functional group variations to the biologics, such as unintended phosphorylation or methylation or even structural changes. These changes could potentially alter the pharmacological activity of the biologic and even increase immunogenicity.

## 3 Asthma

Asthma is the most common chronic inflammatory disease of the lower and upper respiratory tract, characterised by bronchial hyperresponsiveness to a range of factors which leads to bronchoconstriction and reversible expiratory flow limitation. Asthma is a heterogeneous disease with a significant degree of variability of phenotypes (clinical presentations) and endotypes (underlying pathophysiological pathways). Characteristic symptoms include wheezy respiration, shortness of breath, coughing and chest tightness. Symptoms vary in severity and frequency and occur primarily during asthma exacerbations. Bronchial asthma can be distinguished into two major endotypes based on the presence of inflammatory responses driven by T-helper cells type 2 (Th2). The two classically recognised endotypes are type 2 (eosinophilic) asthma and non-type 2 (non-eosinophilic) asthma. The discovery that type 2 innate lymphoid cells (ILC2s) can release Th2 cytokines led to a more precise classification of asthma endotypes into T2-high, T2-low and non-T2 (10).

The current understanding of asthma pathogenesis is that bronchial hyperresponsiveness to normally harmless antigens such as pollens or mites causes epithelial cells to release inflammatory mediators, including several interleukins. The mediators trigger a cascade of immune activation which involves ILC2s, mast cells, Th2s, eosinophils and dendritic cells. Recurring exposure to allergens can lead to chronic airway inflammation and airway remodelling, ultimately resulting in loss of pulmonary function. Airway remodelling in asthma involves subepithelial fibrosis, extracellular matrix deposition, goblet cell proliferation, smooth muscle and mucosal gland hypertrophy and epithelial damage (11). A range of novel biologic agents and small molecule inhibitors is currently undergoing preclinical testing and clinical trials, of which many target only factors responsible for particular endotypes - such as monoclonal antibodies targeting type 2 cytokines (12, 13).

### 3.1 FDA-approved asthma biologics

Cytokines such as IL-4, IL-5, IL-9 and IL-13 and their receptors have been chosen as targets for monoclonal antibody-based treatments due to their importance in the pathogenesis of asthma. Several therapeutic anti-interleukin mAbs have already been approved for the treatment of asthma. Mepolizumab binds IL-5, blocking its effects. The mAb was found to improve lung function and reduce exacerbation frequency in asthmatic patients exhibiting blood eosinophil levels above 150 cells/µL (14, 15).

Benralizumab is an afucosylated anti-IL5-Rα mAb. The antibody induces a strongly enhanced antibody-dependent cellular cytotoxic effect (ADCC) which results in eosinophil depletion. In patients with strong eosinophil-mediated inflammation, benralizumab was found to improve clinical outcomes (16, 17).

Omalizumab is a recombinant humanised monoclonal antibody (mAb) designed to bind free IgE selectively. The specificity-independent binding of IgE by omalizumab inhibits IgE-FcϵRI (high-affinity IgE receptor) interactions, limiting activation of the IgE-mediated allergic inflammatory cascade (18). Omalizumab induces a depletion of free IgE, reducing FcϵRI expression on basophils, mast cells and dendritic cells, which in turn limits the antigen presenting capabilities of those cell types (19, 20). Omalizumab was found to reduce early and late asthmatic responses, improve lung function and exacerbation frequency and severity. A greater therapeutic effect was found in patients exhibiting high circulating eosinophil, periostin and FENO levels (21, 22).

Dupilumab is a fully human anti-IL4-Rα mAb which has been used to treat all type 2 inflammation-related conditions. Usage of the mAb was linked to significant asthma exacerbation rate and oral corticosteroid usage rate reductions, most significantly in patients with high FENO and blood eosinophil levels (23). A summary of the anti-interleukin biologics currently approved by the US FDA for the treatment of moderate to severe type-2 asthma can be found in **Table 2**.

**Table 2:** Summary of anti-IL biologics currently approved by the US FDA for the treatment of moderate to severe type-2 asthma (24).

Therapeutic	Mechanism of action	Effects on asthma exacerbation frequency	Effects on lung function
Omalizumab	Blocks IgE from binding FcϵRI	25% reduction	Minimal
Mepolizumab	Binds IL-5 ligand, blocking IL-5-IL-5R interactions	50% reduction	Inconsistent
Reslizumab	Binds IL-5 ligand, blocking IL-5-IL-5R interactions	50-60% reduction	Significant improvement
Benralizumab	Binds IL-5Rα, blocking IL-5-IL-5R interactions and leading to eosinophil, basophil apoptosis	25-60% reduction	Significant improvement
Dupilumab	Binds IL-4Rα, blocking IL-4 and IL-13 signalling	50-70% reduction	Significant improvement

### 3.2 Asthma biologics undergoing clinical trials

#### 3.2.1 TSLP

Thymic stromal lymphopoietin (TSLP) is a type I cytokine of the IL-2 family. TSLP activity amplifies the Th2 immune response and promotes a shift from immune tolerance to pro-inflammatory signalling pathways (25). Cells in asthmatic airway epithelial and submucosal tissues express TSLP at a markedly increased rate, as compared to healthy tissues (26). What is more, TSLP expression remains increased in severely asthmatic patients, despite high-dose corticosteroid treatments (27). These factors make TSLP an attractive therapeutic target in asthma (28).

Multiple anti-TSLP mAbs have been in development, but most, such as ASP7266 (29) or RG7258 (30) have been discontinued in early clinical trials. Tezepelumab (AMG 157, MEDI 9929) was the first anti-TSLP mAb to be approved by the US FDA for asthma treatment. Tezepelumab is a fully human anti-TSLP IgG2λ mAb. Binding of TSLP by tezepelumab blocks TSLP-receptor interaction and results in inhibition of inflammatory pathways activated by TSLP-mediated signalling (31).

A phase I trial (32) was conducted on the effects of intravenous tezepelumab (700 mg dose) administered once per 4 weeks, over 12 weeks to patients with mild allergic asthma, after an induced allergen challenge. A reduction in airway hyperresponsiveness, circulating eosinophil levels (59% reduction in the tezepelumab group vs 21% in the placebo group), FeNO and sputum eosinophil levels was seen in patients treated with the mAb. Both the early and late asthmatic responses were significantly reduced in the tezepelumab groups, with reductions of 31% for the early response and 46% for the late response, as compared with the placebo group (32). A phase II randomised, double blind, placebo-controlled trial (33) was subsequently conducted. Asthma patients undergoing inhaled corticosteroid or long acting β2-agonist therapy were assigned one of three dose levels of subcutaneous tezepelumab or placebo. Non-placebo group patients were administered 70 mg once per 4 weeks (low dose group), 210 mg once per 4 weeks (medium dose group) or 280 mg once per 2 weeks (high dose group) over a period of 52 weeks.

After 52 weeks, the annualised exacerbation rate for the low dose group was reduced by 61%, by 71% for the medium dose group and by 66% for the high dose group. These reductions were independent from changes in blood eosinophil counts, serum IgE levels and FeNO. A significant increase of FEV1, improvements in symptom control and self-reported quality of life were also reported (33). A subsequent phase III trial (34) demonstrated 210 mg of tezepelumab administered once per 4 weeks to be the optimum dose for severe asthma patients. The same study also determined that tezepelumab-mediated reductions of exacerbation rates and FeNO are independent from biomarkers of type 2 inflammation (34).

Following these results, the US FDA granted tezepelumab breakthrough drug status for use in severe asthma patients, making it the first anti-TSLP mAb used in asthma therapy. Recently, tezepelumab, sold by AstraZeneca under the name Tezspire, has been approved for severe asthma treatment in the European Union. Importantly, Tezspire is suitable for patient self-administration, removing the need for hospital visits (35). Since then, research on several other anti-TSLP biologics has begun, but to date none have been approved.

A TSLP-binding IgG1/λ Fab fragment named ecleralimab (formerly CSJ117) has undergone a Phase I randomised clinical trial (NCT03138811) (36). The trial aimed to assess safety, pharmacokinetics and pharmacodynamics and tolerability of the Fab fragment administered via inhalation, in mild asthma patients upon exposure to allergen challenge. One of three unspecified doses were delivered once daily to the trial subjects, over a period of 12 weeks. The Fab fragment was found to be safe and well tolerated. What is more, ecleralimab was reported to reduce allergen-induced bronchospasms (36). Based on these results, the mAb has entered a phase IIb multicentre, multi-national, double-blind, randomized, parallel-arm, placebo-controlled trial (NCT04410523) (37) involving subjects with asthma which is uncontrolled, despite medium-to-high dose ICS and LABA therapy. As of November 2022, recruitment for this trial has been terminated by sponsor decision, and no results or public statements regarding ecleralimab have since been published. The study aimed to compare the effects of 5 different ecleralimab doses added to standard of care to placebo in approximately 625 patients (37).

TQC2731 is a humanized anti-TSLP monoclonal antibody. TQC2731 is currently undergoing a Phase II, multicentre, double-blind, randomized, parallel group, placebo-controlled clinical study (NCT05472324) (38). Three doses of TQC2731 will be administered to approximately 220 adult subjects with poor control of severe asthma. The study aims to evaluate the effects of TQC2731 on annualised asthma exacerbation rates. TQC2731 (70 mg, 210 mg, 420 mg every four weeks) or placebo (every four weeks) will be administered via subcutaneous injection. The study is slated to be completed in October 2024 (38).

Cytokine traps are fusion proteins consisting of an IgG constant region and extracellular domains of two distinct cytokine receptor components which bind a specific cytokine. TSLP-traps, consisting of TSLPR and IL7-Ra ectodomains have been developed as a novel approach to TSLP inhibition. These fusion proteins were found to neutralise TSLP with a KD three orders of magnitude higher than their non-fusion counterparts alone. What is more, TSLP-trap inhibition efficacy of TSLP-induced STAT5 signalling was found to be 20-fold higher than that of tezepelumab. TSLP-traps were also found to inhibit TSLP-mediated dendritic cell activation with a potency comparable to tezepelumab (39). Despite these promising *in vitro* results, no TSLP-trap-based therapeutics are currently in development. Th2 asthma patients often present with disease phenotypes resistant to steroid therapeutics, with most novel therapies eliciting negligible effects in those patients. It is hypothesised that this lack of efficacy may stem from only targeting single redundant inflammatory pathways, whilst others remain unaffected. To solve this issue, Venkataramani et al. have developed novel bispecific antibodies called Zweimabs and Doppelmabs. The bispecific antibodies bind both TSLP and IL-13 at very high affinities. As stated before, TSLP and IL-13 are attractive targets for asthma therapeutics due to their importance in asthmatic cellular signalling and overlap of the signalling effects of both cytokines (40). What is more, both TSLP and IL-13 are co-expressed in the lung epithelial tissues of severe asthma patients (41). The novel bispecific antibodies were found to have stronger affinities to human cellular targets than their parental monospecific antibodies. Despite these highly promising initial results, no clinical studies involving zweimabs or doppelmabs have been announced so far (40).

### 3.3 Other therapeutic targets

Depemokimab (GSK3511294) is an anti-IL-5 mAb currently undergoing clinical trials conducted by GlaxoSmithKline. The mAb has been engineered to extend half-life and improve IL-5 binding affinity. In 2021, results of a double-blind, parallel-group, single-ascending-dose, multicentre, Phase 1 clinical trial (NCT03287310) (42) were published. Asthma patients with blood eosinophil counts >200 cells/μL were recruited and randomised between dose and placebo cohorts. Patients in the dose cohorts were administered a single subcutaneous dose of depemokimab. After 40 weeks, endpoints were measured. 92% of patients in the placebo group experienced adverse asthmatic events, as opposed to 81% in the mAb-treated group. Reductions in blood eosinophil counts of 48% or more were seen 24 post-dose in the mAb groups, but not in the placebo groups. Pharmacokinetics of the mAb were linear and dose-proportional. In particular, the duration of blood eosinophil count reductions was proportional to the dose administered. At six months post-dose, compared to placebo, depemokimab 2 mg induced a blood eosinophil reduction of 31%, and depemokimab 300 mg - a reduction of 83% (42). Based on these results, a further, phase 3 trial (NCT04718103) (43) investigating the safety profile and efficacy of depemokimab is being conducted. The study is estimated to complete in May of 2024 (43). Most other IL-5 inhibitors are administered every 4 or 8 weeks, while depemokimab is to be administered every 6 months. Such a long-lasting effect allowing infrequent administration would likely increase patient adherence to the therapy while lowering the risk of adverse reactions.

Interleukin 8 (IL-8) is a human chemokine and the strongest chemotactic factor in humans. It is produced by macrophages and several types of epithelial cells. IL-8 acts through the CXCR1 and CXCR2 chemokine receptors on neutrophils, T lymphocytes, monocytes, macrophages and basophils. Interleukin-8 works by stimulating the migration of neutrophils, monocytes and T cells; it causes the adhesion of neutrophils to the endothelium and the release of histamine from basophils. In addition, it also stimulates angiogenesis. In asthma, increased sputum IL-8 levels are often found preceding asthma exacerbations and coincide with progression of airway constriction in patients with coexisting atopic allergies (44). IL-17A and IL-17F produced by Th17 lymphocytes induce production of IL-8 by epithelial cells. CJM112 is an anti-IL-17A monoclonal antibody. A Phase II clinical trial (NCT03299686) (45) of CJM112 recruited patients with uncontrolled moderate-to-severe asthma, low serum IgE and low blood eosinophil counts. CJM112 did not improve FEV1 compared to placebo, but statistically significant improvements in ACQ6 and ACQ7 (asthma control questionnaire) scores were observed in the CJM112 group compared with placebo (13, 45).

CM310 is a humanized, highly potent antibody against IL-4Rα, being developed as a treatment for a wide range of type II allergic diseases including moderate-to-severe eosinophilic asthma. It is currently undergoing a Phase IIb clinical trial (NCT05186909) (46) in subjects with moderate-to-severe asthma to evaluate the efficacy, safety, PK characteristics, PD effects and immunogenicity of the mAb (46). In an annual results presentation by Keymed Biosciences (47), CM310 was claimed to inhibit T cell activation *in vitro* at a potency comparable or higher to competitors, such as dupilumab. CM310 was also said to inhibit the IL-4 or IL-13-induced phosphorylation of STAT6 more effectively than Dupilumab and inhibit IL-4 or IL-13 induced proliferation of TF-1 cells with similar or higher potency to Dupilumab. However, these claims are not insofar supported by peer-reviewed studies and appear only in company-published documents (47).

CM326, another biologic asthma therapeutic under development by the same company, has undergone a Phase I clinical trial (NCT04842201) (48). CM326 is an anti-TSLP monoclonal antibody. Although no results have been posted in the first clinical trial, the mAb is currently undergoing a further clinical trial (NCT05171348) to evaluate the safety, tolerability, pharmacokinetics and immunogenicity of multiple ascending doses of subcutaneously administered CM326 in healthy subjects (49).

Interleukin 33 (IL-33) is an alarmin released by airway epithelial cells, airway smooth muscle cells and endothelium in response to cellular damage or allergen exposure. IL-33 signalling triggers both innate and adaptive immune responses (50). IL-33 was found to activate type 2 innate lymphoid cells and promote eosinophilia in severely asthmatic patients (51). Thus, IL-33 and IL-33 receptor (IL-33R) are attractive candidates for inhibition in asthma therapy.

Itepekimab is a monoclonal antibody directed against IL-33. The results of a phase II randomized clinical trial (NCT03387852) (52) of its use in patients with moderate to severe asthma were published in late 2021. Adult patients receiving inhaled steroids plus additional long-acting beta-adrenergic agonists were equally randomized into one of four treatment arms: itepekimab 300 mg, itepekimab and dupilumab 300 mg each, dupilumab 300 mg alone, or placebo. The treatments were administered every 14 days for 12 weeks. After randomization, treatment with long-acting beta-adrenergic agonists was discontinued and the dose of inhaled corticosteroids was reduced at weeks 6 and 9 of treatment. The primary endpoint was the occurrence of an event suggesting asthma exacerbation.

A total of 296 patients were randomized. By week 12, 22% of patients in the itepekimab arm exhibited asthma exacerbations, compared to 27% in the itepekimab and dupilumab arm, 19% in the dupilumab arm and 41% in the placebo arm. There were no statistically significant differences in the incidence of adverse events between the study arms (52). Significant improvements in FEV1 compared to placebo were seen in the itepekimab arm and dupilumab arm of the study, but not in patients treated with combination therapy. Itepekimab, both alone and in combination therapy, improved patient ACQ5 scores compared to placebo, to a degree similar to dupilumab alone. Blood eosinophil counts compared to placebo were significantly lowered in the itepekimab and combination therapy arms of the study, but not in the dupilumab arm. Dupilumab monotherapy induced transient eosinophilia in some patients, which is consistent with previously published results (28, 53).

A summary of biologics currently undergoing clinical trials in the treatment of asthma can be found in **Table 3**.

**Table 3:** Summary of biologics currently undergoing clinical trials in the treatment of asthma.

Drug	Target	Biological effects	Clinical effects	ClinicalTrials.gov trial identifiers
TQC2731	TSLP	None published	Safe and well tolerated in Phase I trial (54)	NCT05472324 (38)
Depemokimab	IL-5	2x reduction in clearance rate and, 30x higher IL-5 binding affinity compared to mepolizumab (42)	Significant reductions in adverse event rates and blood eosinophil counts in Phase I trial (42)	NCT04718103 (43)
CJM112	IL-17A	10x higher affinity to IL-17A and 200x higher affinity to IL-17AF compared to secukinumab (55)	Significant improvements in ACQ6 and ACQ7 scores in Phase II trial (45)	NCT03299686 (13, 45)
CM310	IL-4R	More efficient inhibition of IL-4 and IL-13 phosphorylation of STAT6 than dupilumab and comparable inhibition of TF-1 cell proliferation to dupilumab (47)	None published	NCT05186909 (46)
CM326	TSLP	None published	None published	NCT05171348 (49)
Itepekimab	IL-33	None published	Significant asthma exacerbation rate reductions, significant FEV1 improvements, significant reductions in blood eosinophil counts and improvements in ACQ scores in a Phase II trial (52)	NCT03387852 (52)

Eosinophilic airway inflammation in severe asthma has successfully been recognised as treatable by biologic drugs. Anti-IL5, anti-IL4R and anti-IgE mAbs have been used to improve outcomes from patients suffering from T2-high asthma endotypes. Most asthma patients however, do not require biologic therapies as adherence to oral corticosteroid (OCS) therapy is frequently sufficient to manage symptoms. Nevertheless, biologic treatments may be of critical importance for those patients who do not tolerate or respond to OCS treatments and exhibit eosinophilic T2-high asthma endotypes. Importantly, no efficacious biologic treatments are available for patients with T2-low or neutrophilic asthma. No specific biomarkers for T2-low asthma are currently known and pathomechanics of this endotype are not well understood. However, several neutrophilic airway inflammation-related targets for drug development were identified and are currently being investigated, including IL-1, IL-6, IL-8, IL-17, IL-23 and TNF-α (13). Further studies are required to characterise T2-low asthma pathogenesis with more detail and create foundations for the development of effective biologic treatments.

## 4 Chronic obstructive pulmonary disease

Chronic obstructive pulmonary disease (COPD) is a chronic, progressive inflammatory lung disease and a cause of major and rapidly growing social and economic burdens worldwide. It is predicted to become the third top cause of death globally (56). COPD is characterised by small airway impediment and destruction of lung parenchyma resulting in aberrant lung function and unremitting airflow limitation (57). The precise pathogenesis underlying COPD is not yet fully understood, but mechanisms such as oxidative stress, protease-antiprotease imbalance and inflammation triggered by a range of pollutants and tobacco smoke are believed to be key risk factors (58–60). Smoking of tobacco-based products is known to be the leading cause of COPD. Smoking induces chronic inflammation in lung tissues, causing inflammatory cell infiltration into lung epithelium. Factors released by immune cells cause persistent irritation of functional lung tissue. While repeated tissue repair at inflammation sites leads to collagen deposition and ultimately - thickening of bronchial walls and narrowing of small airways. As the disease progresses, the ability of the lungs to produce extracellular matrix (ECM) is reduced, and the protease - antiprotease balance is lost, resulting in emphysema and lung parenchyma degradation (61–63). COPD-related inflammation is principally neutrophilic, but 20 to 40% of patients exhibit eosinophilic inflammation. Rarer subgroups of patients may also be affected by a combination of neutrophilic and eosinophilic inflammation, with varying proportions (64). Other immune cells, such as macrophages, dendritic cells, B cells and T cells, and epithelial cells may likewise take part in COPD inflammation. In COPD, these cell types produce an intricate system of mediators, including cytokines, ROS and proteases (65). Patient-specific disease subtypes likely vary based on the underlying mechanisms of pathogenesis and proportion of contributions of neutrophil-associated (T1) and eosinophil-associated (T2) inflammation (64, 66).

Much attention has been given to the idea of therapeutics able to modulate chronic lung inflammation in COPD, as most airway pathologies in COPD respond poorly to corticosteroid therapy (67). The primary aims of COPD drug research are therefore to develop agents capable of either inhibiting COPD-mediating inflammatory cell recruitment and activation directly, or indirectly - by targeting inflammatory mediators and blocking them from interacting with inflammatory cells. However, patients with COPD are at increased risk of persistent and severe lung infections. This increased risk may be further exacerbated by such drugs, which have the potential to impair patient immune responses (65, 68). The two primary pharmaceutical approaches to COPD management are bronchodilators such as β2-adrenergic agonists or antimuscarinics and anti-inflammatory drugs such as corticosteroids. As neutrophilic inflammation is present in most COPD cases, the first attempts at developing biologics for COPD therapy have focused on targeting the mechanisms of T1 inflammation. However, the efficacies of these first attempts were less than satisfactory, and adverse reactions were common (69).

Attempts at safe and effective mAb-mediated CXCR2 inhibition (70) and TNF-α inhibition (71) have also been unsuccessful, with high incidence of adverse effects and no improvements in patient health found in clinical trials. Thus, further attempts at COPD biologics have turned their attention to primarily treating COPD-related eosinophilia (72). Recruitment and activation of immune cells have been successfully inhibited *in vitro* with use of several classes of drugs, including phosphodiesterase inhibitors, chemokine receptor inhibitors, p38 MAPK inhibitors, PI3K inhibitors and anti-IL17A mAbs (73, 74). Several anti-IL17A mAbs are being developed as potential COPD therapeutics. However, certain anti-IL17A mAbs such as CNTO 6785 have been shown to exacerbate COPD-related bacterial infections in patients, while several others such as COVA322, NI-1401, secukinumab or brodalumab are yet to be tested on COPD patients (73). Due to a percentage of COPD patients exhibiting T2 inflammation, anti-IL5 and anti-IL4 mAbs are also being researched for COPD. Mepolizumab and benralizumab, previously approved for asthma treatment, have been shown to reduce exacerbation rates in COPD patients exhibiting eosinophilia (73, 75). As described earlier IL-4, IL-13 and TSLP are known to be drivers of T2 inflammation. Due to this connection, dupilumab, an anti-IL4Rα mAb approved for asthma treatment, and tezepelumab an anti-TSLP mAb also approved for asthma treatment are also being tested in COPD patients with eosinophilia via randomised clinical trials (76–78).

METREX (NCT02105948) (79) and METREO (NCT02105961) (80) are two phase III clinical trials of mepolizumab in moderate to severe COPD which concluded in 2018. 836 subjects recruited for METREX were randomised into a 100 mg mepolizumab arm or placebo arm at a 1:1 ratio, and received respective treatments every 4 weeks for 52 weeks. 674 subjects in METREO were randomised to receive 100 mg or 300 mg mepolizumab, or placebo at a 1:1:1 ratio, also every 4 weeks over 52 weeks. Subjects were grouped into eosinophilic and non-eosinophilic disease endotypes based on blood eosinophil count screenings and blood eosinophil count history over the previous year. METREX included both disease endotypes, while METREO examined only patients with eosinophilic inflammation. A pre-planned *post hoc* analysis of both studies found that annual rates of disease exacerbation in patients with eosinophilia treated with 100 mg mepolizumab were 18 to 20% lower than in the placebo arm. 300 mg of mepolizumab was not found to bring about any additional reduction in exacerbation rates or other benefits, as compared to the 100 mg dose. No lung function improvements or adverse events were found in the mepolizumab arm, as compared to placebo. Importantly, non-eosinophilic patients who received mepolizumab had poorer clinical outcomes than those in the placebo arm, underlining the importance of correctly identifying eosinophilia before introducing anit-T2 inflammation biologic treatments (81).

Benralizumab under the trade name Fasenra is currently being tested for COPD in the AstraZeneca-funded Phase III RESOLUTE trial (NCT04053634) (82). The study aims to evaluate the efficacy and safety of benralizumab in patients with moderate to very severe COPD with a history of frequent COPD exacerbations and elevated peripheral blood eosinophils. 642 participants will be recruited and randomised 1:1 to either the placebo arm or 100 mg benralizumab arm of the study. Both treatments will be administered subcutaneously every 4 weeks for the first 3 doses, then every 8 weeks over a period of 56 weeks. The primary measured endpoint for this trial is the annualised rate of moderate or severe COPD exacerbations measured over the time of the trial. The results of the RESOLUTE trial are to be published in 2025 (82).

Interleukin 1 receptor-like 1, also known as IL1RL1 and ST2, is a protein involved in IL-33 signalling which has been shown to be a driver of inflammatory responses. The direct role of ST2/IL-33 signalling is unclear, though IL-33 itself is known to mediate eosinophil maturation and eosinophil recruitment to the airways in chronic inflammatory lung diseases (83).

Astegolimab is an anti-ST2 mAb which has been tested in a randomised Phase IIa clinical trial named COPD-ST2OP (NCT03615040) (84). COPD-ST2OP was a single-centre, double-blind, placebo- controlled, parallel group, randomised controlled trial to assess the efficacy and safety of anti-ST2 compared to placebo, in patients with moderate to very severe COPD. The trial was completed in August 2020. 81 participants were randomised to receive either 490 mg of asteligomab subcutaneously or placebo, every 4 weeks for 44 weeks. Astegolimab treatment within the clinical trial did not reduce exacerbation rates as compared to placebo, however it did improve patient health status as measured by eosinophil counts, FEV1 and St. George’s Respiratory Questionnaire for COPD scores (83).

These results served as the basis for further Phase IIb (NCT05037929) (85) and Phase III (NCT05595642) (86) randomised clinical trials at a much larger scale, each recruiting 1290 patients. This pair of astegolimab clinical trials is slated to complete in 2025.

TITANIA (NCT05158387) (87) and OBERON (NCT05166889) (88) are a pair of parallel Phase III randomised clinical trials evaluating the efficacy and safety of tozorakimab administered subcutaneously in adult participants with symptomatic COPD and a history of COPD exacerbations in the previous 12 months. Tozorakimab is an anti-IL-33 mAb which has shown a favourable safety and pharmacokinetics profile in earlier, small scale clinical trials (NCT04631016) (89). This pair of clinical trials aims to recruit 1272 participants for each trial with the primary measured outcome being the annualised rate of moderate to severe COPD exacerbations in participants who are former smokers. The results of these clinical trials are to be published in late 2025 (87, 88).

A summary of biologics currently undergoing clinical trials in the treatment of COPD can be found in **Table 4**.

**Table 4:** Summary of biologics currently undergoing clinical trials in the treatment of COPD.

Drug	Target	Biological effects	Clinical effects	Other FDA-approved indications	ClinicalTrials.gov trial identifiers
Mepolizumab	IL-5	Significant reductions in eosinophil activation and increases in IL-5 and IL-5 receptor expression levels ex vivo (90)	Significant reductions in annual disease exacerbation rates in Phase III trials (81)	Severe eosinophilic asthma, inadequately controlled CRSwNP, Churg-Strauss syndrome, hypereosinophilic syndrome	NCT02105948 (79), NCT02105961 (80)
Benralizumab	IL5-Rα	Rapid depletion of peripheral eosinophil count in cynomolgus monkeys (91)	Did not significantly reduce exacerbation and adverse event rates in previous COPD studies (92, 93)	Severe eosinophilic asthma	NCT04053634 (82)
Astegolimab	ST2	None published	Significant improvements in QoL questionnaire scores, FEV1 and reductions in blood eosinophils in Phase IIa trial (84)	None	NCT05037929 (85), NCT05595642 (86)
Tozorakimab	IL-33	Potent inhibition of ST2-dependent inflammation in primary human cells and a humanised IL-33 mouse model of airway inflammation, exhibited 3x higher binding affinity for IL-33 than sST2 (94)	Favourable safety and pharmacokinetics profiles shown in Phase I clinical trial (89)	None	NCT05158387 (87), NCT05166889 (87)

As research in the field of COPD biological therapies progresses, the network of interactions between inflammation, chronic infections and other pathogenetic factors underlying the disease is becoming clearer. Recent developments in the field have provided several valuable and even surprising discoveries such as the apparent inefficacy of targeting neutrophilic inflammation and cytokines with biologic treatments and the increases in adverse event rates related to these treatments. On the flipside, therapies aimed at eosinophilic inflammation have provided far more promising results, albeit not as pronounced as those seen in asthma biologic therapies. These differences in efficacy might be linked to the disparity between mechanics driving eosinophilic inflammation in COPD and asthma (72). Thus far, biologics have not seen widespread usage in COPD. However, once the identification of patient subgroups more likely to benefit from biologics becomes more straightforward, the value of such therapies will become more apparent. Targeting upstream signalling molecules such as TSLP and IL-33, and developing biologics for the management of chronic infections might prove to be especially beneficial for COPD patients.

## 5 Idiopathic pulmonary fibrosis

Idiopathic pulmonary fibrosis (IPF) is a progressive disorder characterised by the accumulation of extracellular matrix (ECM) components in the lungs (95). The progressive accumulation leads to formation of scar tissue and consequently, irreversible loss of lung function (96). Prognosis of IPF is poor, with most diagnoses leading to patient death, with a 5-year survival rate of 20 to 30% (97). Unfortunately, despite the poor prognosis and high impact on global health, no effective IPF treatments currently exist. Several biologic drugs are currently under development as potential IPF therapeutics. IPF pathogenesis is believed to result from a network of interactions between inflammatory cells, fibroblasts and the lung parenchyma. Cytokines released by the inflammatory cells exhibit profibrotic activity, which in the context of chronic inflammation leads to fibroblast proliferation and transformation, ECM accumulation and tissue damage. The exact mechanisms and pathways underlying the pathogenesis of IPF are not fully understood. However, some proteins such as transforming growth factor β1 (TGF-β1) or connective tissue growth factor (CTGF) are known to take part in all fibrotic processes. As such, these proteins are potential targets for IPF therapies (98).

Pamrevlumab (formerly FG-3019) is a mAb which binds CTGF, blocking its activity. CTGF is a protein critical for fibrosis, and as such - the pathogenesis of IPF.

ZEPHYRUS-1 (NCT03955146) (99) is an ongoing Phase III randomised, placebo-controlled clinical trial of pamrevlumab which aims to evaluate the safety and efficacy of one year of treatment with the mAb. Either 30 mg of Pamrevlumab per kg of patient bodyweight or placebo will be administered intravenously every 3 weeks over 52 weeks. ZEPHYRUS-1 includes 356 IPF patients across 144 sites worldwide, and results are to be released in June 2023 (99).

ZEPHYRUS-2 (NCT04419558) (100) is a second Phase III trial which also constitutes a part of the pamrevlumab development program. ZEPHYRUS-2 is currently recruiting 340 IPF patients in between the ages of 40-85, who have previously undergone IPF therapy. The dosage, route and frequency of administration of pamrevlumab in ZEPHYRUS-2 will be the same as for ZEPHYRUS-1 (100). The primary endpoint measurement for both the ZEPHYRUS studies is the assessment of FVC changes in pamrevlumab-treated patients versus placebo. Secondary endpoints include lung fibrosis progression and patient-reported symptom control scores. ZEPHYRUS-1 includes patients who currently are not being treated with any other IPF therapy for a range of reasons including intolerance, ineligibility or concerns about treatment-related risks. On the other hand, ZEPHYRUS-2 aims to assess the efficacy and safety of pamrevlumab in patients who have undergone other IPF therapies, such as pirfenidone (101) or nintedanib (102). Previous Phase II trials (NCT01890265, NCT01262001) (103, 104) of pamrevlumab in mild-to-moderate IPF have shown the therapeutic to be safe and effective. At 48 weeks, patients who received pamrevlumab exhibited a 10% risk of disease progression, versus 31,4% in the placebo arm. FVC reduction was also limited in patients who received pamrevlumab by around 60% versus placebo (103, 104).

MicroRNAs (miRNAs) are non-coding RNAs that play important roles in regulating gene expression. Many miRNAs negatively regulate expression in gene networks. Most miRNAs undergo transcription from DNA to primary miRNAs which are then processed into precursor miRNAs and ultimately mature miRNAs (105). MRG-299 is a miR-29 mimic engineered by Chioccioli et al., based on MRG-201. MRG-299 was modified to improve chemical stability through additional sugar modifications and the addition of an internally conjugated platelet-derived growth factor β receptor (PDGFβR)-binding peptide (106). The researchers investigated the activity of MRG-229 in several *in vivo* studies on animal models. MRG-229 was successful in decreasing the expression of pro-fibrotic pathways and reducing collagen production in all models. Bleomycin-treated mice exhibited a downregulation in profibrotic pathway expression upon MRG-229 treatment, at doses 10 times lower than the original MRG-201 miRNA mimic. Chioccioli et al. also tested MRG-229 on rats and non-human primates. The compound was found to be well tolerated and caused no adverse reactions at clinically relevant doses. Lastly, the researchers examined miR-29 levels in peripheral blood samples from 259 IPF patients. Lower miR-29 levels were found to correlate with increased mortality in the examined cohort. Summarily, these results serve as a basis for the development of MRG-229 and other miRNA mimics as potential therapeutics against IPF (107).

A summary of biologics currently undergoing clinical trials in the treatment of IPF can be found in **Table 5**.

**Table 5:** Summary of biologics for the treatment of idiopathic pulmonary sarcoidosis which are currently undergoing development.

Drug	Target	Biological effects	Clinical effects	ClinicalTrials.gov trial identifiers
Pamrevlumab	CTGF	Consistently diminished fibrotic responses in a novel intratracheal bleomycin instillation animal model of pulmonary fibrosis (108)	Reduction in FEV1 decline and disease progression rate over 48 weeks in a Phase II RCT (109)	NCT03955146 (100), NCT04419558 (100)
MRG-299	miR-29 mimic	Caused a downregulation in profibrotic pathway expression in bleomycin-treated mice (107)	None published	None

## 6 Pulmonary sarcoidosis

Sarcoidosis is a multisystem disorder characterised by the presence of noncaseating granulomas - clusters of immune cells including macrophages, lymphocytes, monocytes and CD4+ T cells (110). The etiology of sarcoidosis is thought to stem from a mixture of genetic predispositions and environmental factors. The precise underlying pathomechanisms of the disorder are yet to be elucidated (111). As the disease manifests in a range of organs with widely varying symptoms and efficacious treatments are lacking, severe sarcoidosis symptom control is very challenging. Thus, the development of new, effective therapies is of high importance.

As stated before, sarcoidosis can affect almost any organ, however in over 90% of cases, symptoms localise to the lungs and intrathoracic lymph nodes (112, 113). Common symptoms of pulmonary sarcoidosis include dyspnea, chest pain, coughing and chest tightness. Importantly, nearly 1 in 2 patients are asymptomatic and overall prognosis of the disease is good. 80% of stage I pulmonary sarcoidosis patients experience spontaneous regression of lung abnormalities. Only 5% of patients develop chronic respiratory impairment over 10 years (114).

Further stages of the disorder present with far poorer prognoses. Only 30% of patients with stage II and stage III pulmonary sarcoidosis experience spontaneous regression of lung abnormalities. Stage III and stage IV patients exhibit a 5-fold higher risk of chronic respiratory impairment, as compared to stage I patients (115). Due to the largely asymptomatic and spontaneously regressive nature of the disease, most pulmonary sarcoidosis patients do not require treatment. Only about one third of patients require oral glucocorticoid treatment. The American Thoracic Society/European Respiratory Society/World Association of Sarcoidosis and other Granulomatous Disorders (ATS/ERS/WASOG) statement suggests that only patients with progressive symptomatic disease, progressive lung function decline and pulmonary infiltration should be put on a systemic therapy regimen (116). Glucocorticoid treatment constitutes the first line of pulmonary sarcoidosis therapy (117) If glucocorticoids are found to be ineffectual, disease-modifying antirheumatic drug (DMARD) therapy is introduced. Methotrexate is the most used and most efficacious DMARD for pulmonary sarcoidosis treatment (118). Leflunomide, hydroxychloroquine, azathioprine and mycophenolate are also used, albeit less commonly due to weaker evidence of efficacy and safety (119). Biologic drugs are only used as third line, last resort therapeutics in pulmonary sarcoidosis, in cases of disease refractory to both glucocorticoids and DMARDS. The primary reason behind this hesitancy to use biologics is the lack of FDA approval for any biologic agents in the treatment of pulmonary sarcoidosis (120, 121).

Infliximab is the biologic drug most used in pulmonary sarcoidosis treatment. Infliximab is a chimeric anti-TNFα mAb, delivered via intravenous infusions (122). A large multicentre, placebo controlled, double blind RCT was conducted to evaluate the efficacy of infliximab in 138 patients with chronic pulmonary sarcoidosis (123). All patients were receiving corticosteroid treatment at enrolment. Patients were randomised to receive either placebo or infliximab over a period of 24 weeks. Patients in the infliximab arm exhibited a 2,5% FVC increase compared to placebo, and a 26% decrease in the size of radiographically measured reticulonodular opacities. However, infliximab administration did not improve dyspnea or quality of life questionnaire scores. There was no difference in adverse event and infusion reaction rates between the infliximab and placebo groups (123). Another RCT evaluating infliximab vs placebo showed a 15.2% FVC improvement in the infliximab arm vs 8.4% in placebo, however due to a small sample size of only 13 patients, the results were not statistically significant (124).

Adalimumab is a human anti-TNF-α mAb. The drug is delivered subcutaneously, which allows patients to self-administer. The dosing regimen of adalimumab in pulmonary sarcoidosis is based on the previously established irritable bowel syndrome regimen. A loading dose is administered and then maintained by weekly or bi-weekly injections. The safety and efficacy of adalimumab were tested in a 2014 trial. The mAb was administered to a small cohort of patients with refractory pulmonary sarcoidosis over a period of 52 weeks. The antibody was found to have acceptable tolerability and efficacy, leading to FVC, Borg scale and 6MWD improvements (125). Another small trial of adalimumab in infliximab-intolerant patients with pulmonary sarcoidosis led to an improvement of organ function in 7 out of 18 patients (126). However, in a multicohort retrospective analysis conducted in Europe, it was found that out of 240 patients treated with adalimumab, 46 developed anti-adalimumab antibodies. This immunogenicity arose despite the mAb being a fully human antibody with no foreign components (127).

Several other biologics have undergone clinical trials in pulmonary sarcoidosis; however, none have successfully attained satisfactory efficacy and safety profiles. Etanercept, a biologic TNF inhibitor has undergone a Phase II clinical trial in pulmonary sarcoidosis patients, however the trial was terminated prematurely due to a high frequency of adverse events (128). Other biologics such as rituximab, golimumab and ustekinumab have failed to show adequate efficacy in pulmonary sarcoidosis (129, 130). The use of TNF inhibitors in sarcoidosis is also linked with a significant risk of adverse effects, most commonly infections and allergic reactions. A large-scale study on the effects of TNF inhibitors in refractory sarcoidosis has shown a staggering rate of adverse events. 52% of examined patients were affected by adverse events. 23% discontinued TNF inhibitor treatment due to treatment-related adverse reactions (131). A different study examining several infliximab and adalimumab clinical trials in rheumatoid arthritis pointed to a correlation between the use of these mAbs and increased occurrence of malignant tumours and severe infections (132). Due to the largely inadequate efficacy and unreliable safety profiles of TNF inhibitors, several groups are currently conducting research on alternative approaches to biologic-mediated pulmonary sarcoidosis treatment.

Granulocyte-macrophage colony-stimulating factor (GM-CSF) is a monomeric glycoprotein which functions as a hematopoietic growth factor. GM-CSF is secreted by fibroblasts, endothelial cells, T-cells and alveolar macrophages (133). The protein is known to exert proinflammatory effects and stimulate stem cells to differentiate into granulocytes. GM-CSF has been shown to participate in the alveolar cytokine network responsible for the development of granulomatous inflammation in pulmonary sarcoidosis patients (134). Increased levels of GM-CSF were also found in bronchoalveolar lavage fluid (BALF) samples taken from pulmonary sarcoidosis patients. Increases in GM-CSF levels were found to correlate with intensified disease activity (135).

Namilumab is a fully human anti-GM-CSF IgG1 mAb (136). The mAb has been successfully used to control symptoms in rheumatoid arthritis (136, 137) and COVID-19 patients (138). However, Namilumab has not achieved FDA approval for these indications.

RESOLVE-lung (NCT05314517) (139) is a double-blind, placebo-controlled Phase II RCT aiming to evaluate the safety and efficacy of Namilumab in chronic pulmonary sarcoidosis patients (139). The trial aims to recruit 100 patients suffering from chronic pulmonary sarcoidosis, but excludes patients with significant pulmonary fibrosis or sarcoidosis associated pulmonary hypertension (SAPH). Trial participants will be randomised into Namilumab and placebo arms. Participants will receive subcutaneous injections of either Namilumab or placebo every 4 weeks over a period of 26 weeks. All patients enrolled in the trial will be offered a 28-week open label extension (OLE) period following the initial 26-week trial (139). FVC changes after 26 weeks of treatment are the primary measured outcome. Secondary outcomes include Namilumab safety and tolerability, quality of life questionnaire score changes and improvements in extrapulmonary sarcoidosis manifestations. Study results are expected to be published in January 2025 (139).

IL-18 is a proinflammatory cytokine secreted by monocytes and macrophages. IL-18 enhances Th1 IFN-γ production (140). IL-18 alone is insufficient to significantly stimulate IFN-γ production and exerts its full effect only in synergy with IL-12 (141). A meta-analysis of 32 studies totalling almost four hundred pulmonary sarcoidosis patients examined the link between IL-18 levels and pulmonary sarcoidosis (142). IL-18 was found to be increased significantly in both BALF and blood samples of sarcoidosis patients, pointing towards a potential role of IL-18 in the pathophysiology of pulmonary sarcoidosis. What is more, pulmonary sarcoidosis disease progression and IL-18 levels were found to be positively correlated (142).

CMK389 is an anti-IL-18 fully human IgG1 mAb. A Phase II double blind, placebo controlled RCT (NCT04064242) (143) evaluating the safety, efficacy and tolerability of CMK389 in patients with chronic pulmonary sarcoidosis is currently recruiting patients (143). The study aims to enrol 66 symptomatic pulmonary sarcoidosis patients undergoing co-medication with methotrexate or azathioprine for ≥ 6 months prior to screening. Exclusion criteria include the presence of significant pulmonary fibrosis or pulmonary hypertension. Patients will be randomised to receive IV infusions of either CMK389 or placebo every 4 weeks over a period of 16 weeks (143). The study is projected to be completed in December 2023. FVC changes after 16 weeks of treatment are the primary measured outcome. Secondary outcomes include CMK389 safety and tolerability, 6-minute walk distance (6MWD) times, and steroid sparing rates (143).

Neuropilins (NRPs) are single-pass transmembrane, non-tyrosine kinase receptors with short cytoplasmic tails. NRPs are dependent on plexins and VEGF receptors for signal transduction. There are two proteins belonging to the Neuropilin family - Neuropilin-1 (NRP1) and Neuropilin-2 (NRP2) (144). Both NRPs are expressed in all vertebrates and are highly homologous (up to 44%). NRPs are multifunctional and are known to play important roles in axon guidance, angiogenesis, lymphangiogenesis, immunity and the pathogenesis of several conditions including malignancies (145). Importantly, high levels of NRPs were found to be expressed in immune cells, including mast cells, macrophages, T cells and B cells (146). NRP2 expression was found to be upregulated in the alveolar macrophages of a neutrophilic asthma model following the introduction of an immune challenge to the airways. This suggests that NRP2 participates in the regulation of immune responses in asthma (147). The role of NRP2 in sarcoidosis is largely unknown, however NRP2 expression was found in sarcoid granulomas (148).

Efzofitimod, previously known as ATYR1923 is a fusion protein composed of a histidyl-tRNA synthetase (HisRS) domain fused to a human IgG1 Fc fragment. Efzofitimod is an immunomodulating agent which binds NRP2 with a high degree of specificity (149). In a Phase II study, Efzofitimod was tested in patients with pulmonary sarcoidosis. The biologic was found to be safe and well tolerated, with no cases of immunogenicity (150). Efzofitimod is currently undergoing a Phase III multicentre double-blind placebo-controlled RCT in pulmonary sarcoidosis patients (NCT05415137) (151). The study aims to enrol 264 patients currently undergoing oral corticosteroid treatment. Participants must suffer from symptomatic pulmonary sarcoidosis - to have a mMRC (Modified Medical Research Council) Dyspnea Scale score of at least 1 and have an impairment of HRQoL due to the disease. Exclusion criteria once again include the presence of significant pulmonary fibrosis, pulmonary hypertension or extrapulmonary sarcoidosis. Importantly, HisRS (one of the two components of Efzofitimod) is a pathogenic antigen in autoimmune myositis - the Jo-1 antigen. Therefore, patients who test positive for the presence of Jo-1 antigen or have had a history of positive Jo-1 antigen test results will be excluded from the trial. Furthermore, trial participants will be screened for the presence of Jo-1 antigen throughout the trial (151). Participants will be randomised into one of three arms - a placebo arm, a 3 mg/kg Efzofitimod arm or a 5 mg/kg Efzofitimod arm. All patients will receive an intravenous infusion of the respective agent every 4 weeks. The change from baseline in mean daily oral corticosteroid (OCS) after 48 weeks will be the primary measured outcome. Secondary outcomes include FVC change from baseline and King’s Sarcoidosis Questionnaire (KSQ)-Lung score change from baseline. The study is to be completed in January 2025 (151).

A summary of biologics currently undergoing clinical trials in the treatment of pulmonary sarcoidosis can be found in **Table 6**.

**Table 6:** Summary of biologics currently undergoing clinical trials in the treatment of pulmonary sarcoidosis.

Drug	Target	Biological effects	Clinical effects	ClinicalTrials.gov trial identifiers
Namilumab	GM-CSF	22E9 - surrogate mouse antibody of namilumab was shown to neutralise GM-CSF, protect cartilage and suppress inflammation in mouse model of arthritis (152)	Successfully employed in control of rheumatoid arthritis and COVID-19 symptoms (136–138)	NCT05314517 (139)
CMK389	IL-18	None published	None published	NCT04064242 (143)
Efzofitimod	NRP2	Treatment with efzofitimod was shown to reduce *P. acnes*-induced granulomatous inflammation and downregulate inflammatory cytokine signalling in an animal model of pulmonary sarcoidosis (149)	In a Phase Ib/IIa RCT, Treatment with efzofitimod was associated with, dose-dependent trends of improvement for steroid reduction, FVCPP, and PRO scores (150).	NCT05415137 (151)

Compared to asthma and COPD, fewer biologic therapies are being developed for IPF and pulmonary sarcoidosis, perhaps due to the lower incidence of the diseases. However, as the understanding of the potential of biologics for other chronic inflammatory diseases of the lung develops, we may see more research on biologics for the treatment of IPF and pulmonary sarcoidosis as well.

## 7 Conclusions

As evident by the large number of ongoing clinical trials, biologic drugs are a category of therapeutic which is likely to see a surge in importance and prevalence. In the treatment of chronic lung inflammation, biologics offer meaningful advantages over currently used small molecule drugs. Some monoclonal antibodies such as mepolizumab and omalizumab are already being used in the treatment of asthma and constitute an invaluable therapeutic option for patients who tolerate corticosteroid treatment poorly. Other biologics such as depemokimab can be administered highly infrequently, even once per 6 months, significantly reducing the risk of adverse effects.

However, despite significant progress in the development of biologic therapies for chronic inflammatory lung diseases, small molecule drugs will certainly remain mainstay anti-inflammatory treatments for the time being. Biological treatments in general still face several challenges such as high manufacturing and development costs and relatively high risks of immunogenicity. The most optimal future of anti-inflammatory therapy would entail the gradual development of a diverse and complementary library of drugs containing both small molecule drugs and biologic agents.

## Author contributions

Conceptualization, JP and MP; investigation, JP; writing—original draft preparation, JP; writing—review and editing, MP and PK; supervision, MP and PK. All authors contributed to the article and approved the submitted version.
